# Assessment of the dynamics of atrial signals and local atrial period series during atrial fibrillation: effects of isoproterenol administration

**DOI:** 10.1186/1475-925X-3-37

**Published:** 2004-10-22

**Authors:** Luca T Mainardi, Valentina DA Corino, Leonida Lombardi, Claudio Tondo, Massimo Mantica, Federico Lombardi, Sergio Cerutti

**Affiliations:** 1 Department of Biomedical Eng., Polytechnic University of Milan, Via Golgi 39, 20133 Milano Italy; 2 Cardiologia, Dipartimento di Medicina, Chirurgia e Odontoiatria, Osp. San Paolo, Università di Milano, via A. di Rudini 8, 20142 Milan, Italy; 3 Electrophysiology Laboratory, S Ambrogio Hospital, Milan, Italy

## Abstract

**Background:**

The autonomic nervous system (ANS) plays an important role in the genesis and maintenance of atrial fibrillation (AF), but quantification of its electrophysiologic effects is extremely complex and difficult. Aim of the study was to evaluate the capability of linear and non-linear indexes to capture the fine changing dynamics of atrial signals and local atrial period (LAP) series during adrenergic activation induced by isoproterenol (a sympathomimetic drug) infusion.

**Methods:**

Nine patients with paroxysmal or persistent AF (aged 60 ± 6) underwent electrophysiological study in which isoproterenol was administered to patients. Atrial electrograms were acquired during i) sinus rhythm (SR); ii) sinus rhythm during isoproterenol (SRISO) administration; iii) atrial fibrillation (AF) and iv) atrial fibrillation during isoproterenol (AFISO) administration. The level of organization between two electrograms was assessed by the synchronization index (S), whereas the degree of recurrence of a pattern in a signal was defined by the regularity index (R). In addition, the level of predictability (LP) and regularity of LAP series were computed.

**Results:**

LAP series analysis shows a reduction of both LP and R index during isoproterenol infusion in SR and AF (R_SR _= 0.75 ± 0.07 R_SRISO _= 0.69 ± 0.10, p < 0.0001; R_AF _= 0.31 ± 0.08 R_AFISO _= 0.26 ± 0.09, p < 0.0001; LP_SR _= 99.99 ± 0.001 LP_SRISO _= 99.97 ± 0.03, p < 0.0001; LP_AF _= 69.46 ± 21.55 LP_AFISO _= 55 ± 24.75; p < 0.0001). Electrograms analysis shows R index reductions both in SR (R_SR _= 0.49 ± 0.08 R_SRISO _= 0.46 ± 0.09 p < 0.0001) and in AF (R_AF _= 0.29 ± 0.09 R_AFISO _= 0.28 ± 0.08 n.s.).

**Conclusions:**

The proposed parameters succeeded in discriminating the subtle changes due to isoproterenol infusion during both the rhythms especially when considering LAP series analysis. The reduced value of analyzed parameters after isoproterenol administration could reflect an important pro-arrhythmic influence of adrenergic activation on favoring maintenance of AF.

## Background

Atrial Fibrillation (AF) results from multiple, rapidly changing and spatially disorganized activation wavelets sweeping across the surface of the atria [1]. Among factors contributing to genesis and / or maintenance of circulating wavelets, Autonomic Nervous System (ANS) seems to play a major pro-arrhythmic role [2]. The arrhythmogenic influence of sympathetic and vagal mechanisms has been documented in several clinical and experimental studies [3,4]. In men, ablation of the major parasympathetic pathways to the atria drastically reduced vagally mediated atrial fibrillation [4]. It has also been reported that sympathetic stimulation by shortening atrial refractory periods, may increase vulnerability to atrial fibrillation in different experimental models [5]. The shortening of action potential duration and flattening of the restitution slope to cycle length changes induced by adrenergic activation, are two of the mechanisms favoring spiral wave induction and restraining spiral wave break-up [6]. Changes in action potential may also contribute to the perpetuation of atrial fibrillation [7].

In normal hearts, both vagal and sympathetic mechanisms have been associated with paroxysmal atrial fibrillation (PAF) initiation. Most of PAF episodes observed in patients with structural heart disease are triggered by sympathetic activation and vagal withdrawal [8].

Spectral analysis of heart rate variability before PAF episodes has further clarified the pro-arrhythmic role of the autonomic nervous system [9,10]. Bettoni [9] observed a primary increase in adrenergic drive occurring over at least 20 minutes before onset of PAF episodes followed by a shift towards a vagal predominance immediately before arrhythmia onset. Other authors described an increase in sympathetic modulation of sinus node (or a loss of vagal modulation) before PAF onset in the majority of patients [10-12]. More recently Lombardi [7] reported that signs of sympathetic activation characterized up to 70% of PAF episode onset, whereas in the remaining ones a vagal predominance was detectable. An increase in vagal modulation can also promote the stability of AF [13].

Even if AF has been classically described as a random process, a few studies have recently documented, using various signal processing methods, the existence of some determinism underlying AF. Linear analysis techniques documented relationships between intra-atrial recordings using both time-domain methods [14] and spectral-domain approaches [15,16], while the presence of non-linear patterns have been also recognized [17,18]. By using linear and non-linear indexes we have recently assessed [19] the dynamics of intra-atrial signal and local atrial period (LAP) series during different AF episodes. In particular, regularity (R) and synchronization (S) indexes [20], based on the estimation of the corrected conditional entropy and the corrected cross-conditional entropy respectively, were used to describe the dynamics in intra-atrial signals, whereas the LAP series were investigated using regularity and the level of predictability (LP). These parameters were suitable to describe the fine changing characteristic of atrial signals and LAP series [19] when passing from different atrial rhythms classified according to the Wells' criteria [21]. In the present paper, we evaluated whether changes in adrenergic control mechanisms could influence determinisms and dynamics of atrial signals and exploited the capability of linear and non-linear parameters (R and S indexes for intra-atrial signals, R and LP indexes for LAP series) to capture them. Adrenergic activation was mimicked by isoproterenol infusion. The effects of this sympathomimetic drug was evaluated in a small group of patients with a history of PAF during sinus rhythm and atrial fibrillation: four experimental conditions were analyzed (sinus rhythm (SR), sinus rhythm during isoproterenol administration (SRISO), atrial fibrillation (AF) and atrial fibrillation during isoproterenol administration (AFISO)).

## Experimental protocol

### Patient population

Nine patients (8 males and 1 female; mean age 60 ± 6 years) selected to sustain a left atrial ablation with encirclement of the pulmonary veins by transeptal approach were included in the study. All subjects were suffering from atrial fibrillation (AF) and were non responsive to anti-arrhythmic therapy (pharmacological therapy and electrical cardioversion). Paroxysmal and persistent AF episode were present in, respectively, 5 and 4 subjects. A history of AF was present for an interval ranging from 2 months to 10 years. The mean left ventricular ejection fraction was > 40% in all patients; the mean left atrial diameter was 37 ± 3 mm in 7 patients and 51 ± 8 mm in 2. Structural heart disease was present in 4 patients. Reported symptoms included palpitations (6 subjects), fatigue after effort (9 subjects) and syncope (2 subjects). Arterial hypertension was the most common comorbidity in our study group (4 patients). All the patients were in anti-arrhythmic drug wash-out at the time of the study. Flecainide, propafenone, metoprolol, cordarone and methyldigoxin were ceased ≥ 5 half-lives before ablation. Transoesophageal echocardiography was performed the day before the procedure to exclude atrial thrombus.

The Medical Ethical Committee approved this study and all subjects gave their written consent.

### Study design

We investigated the effect of adrenergic activation induced by infusion of isoproterenol on atrial electrical activity. The electrophysiological procedure was performed in the Electophysiology Laboratory of the "Istituto Clinico Sant'Ambrogio" of Milan, Italy. Intracavitary electrocardiograms were recorded during the ablation procedure in which arrhythmic foci inside the pulmonary veins of the left atrium were electrically isolated.

**Figure 1:**
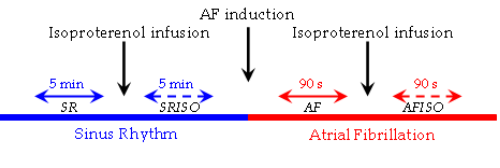
**Timing and sequences of the experimental protocol epochs. **The experimental protocol included four recording periods during: I) sinus rhythm (SR); II) sinus rhythm during isoproterenol infusion (SRISO); III) atrial fibrillation (AF); IV) atrial fibrillation during isoproterenol infusion (AFISO). The recordings during SR lasted at least 5 minutes (range 5 – 8 minutes), those during AF 90 seconds on average (range 60 – 120 seconds). The recordings during infusion of isoproterenol started after the drug had determined a 30% increase of heart rate. Induction of AF started 15 minutes after the end of SRISO, to guarantee the correct isoproterenol wash-out.

The research project protocol included an intracavitary recording of multiple atrial electrograms during sinus rhythm and after induction of atrial fibrillation. In both experimental conditions, the recording was repeated during intravenous infusion of isoproterenol (0.01–0.02 mcg/kg/min) tiered to determine a 30% increase of heart rate. The four clinical experimental conditions were defined as sinus rhythm (SR), sinus rhythm during isoproterenol administration (SRISO), atrial fibrillation (AF) and atrial fibrillation during isoproterenol administration (AFISO). Details on the four epochs of the study can be found in Figure 1.

In all the patients, AF inducibility was obtained at twice diastolic threshold by burst atrial pacing (5'-second bursts at an output of 20 mA) from the mid coronary sinus beginning at a cycle length of 250 ms and reducing by 10 ms intervals until atrial refractoriness. All the nine patients were inducible. AF was considered inducible if it persisted for more than 1 minute. If AF terminated after less than 1 minute, induction was repeated until a maximum of 3 times. If AF became sustained (lasting > 10 minutes), ablation was performed after external DC cardioversion. All our patients underwent the procedure in spontaneous SR.

The duration of registration was an important parameter for the reliability of the analysis, because an insufficient number of atrial potentials (less than 250 – 300) could give errors in the estimation of conditioned probability. Therefore at least 5 minutes (range 5 – 8 minutes) of sinus rhythm and 90 seconds (range 60 – 120 seconds) of atrial fibrillation were registered.

The electrophysiological study was carried out using a deflectable 20 pole *St Jude *catheter (length 95 cm, 7 F, interelectrode spacing 2 – 10 mm), a deflectable decapolar catheter with a distal ring configuration, *Lasso-Cordis Biosense Webster *catheter (length 115 cm, 7 F, interelectrode spacing 2 – 5 mm) and 4 mm distal electrode catheter, *Medtronic Sprinklr*, with irrigated tip (for ablation, length 115 cm, 7 F, interelectrode spacing 2 – 5 mm). The St Jude catheter was in contact with the right atrial wall and inserted in the coronary sinus below the left atrium. The Lasso-Cordis Biosense Webster catheter and the Medtronic Sprinklr catheter were positioned in the superior pulmonary veins at the inside of the atrium.

For the purpose of this study, one surface ECG tracing and nine intracavitary atrial electrograms were stored on digital memory for subsequent off line analysis. In all patients, electrograms labeled 2 – 3 – 4 – 5 corresponded, respectively, to the superior, middle, middle inferior and inferior wall of the right atrium; electrogram 6 to coronary sinus ostium; electrograms 7 – 8 – 9 indirectly corresponded to the inferior and the left wall of the left atrium whereas electrogram 10 to the left superior pulmonary vein.

Electrograms 2 – 9 were recorded with 20 pole *St Jude *catheter, electrogram 10 with *Lasso-Cordis Biosense Webster *catheter.

## Methods and Data analysis

### Regularity

Conditional entropy (*CE*) may be used to estimate a regularity index, defined as the degree of recurrence of a pattern in a signal. *CE *represents the amount of information carried by the most recent sample *x*(*i*) of a normalized realization of *x *when its past *L *- 1 samples are known. *CE *is defined as [22]:



where *p*(*x*_*L *- 1_) represents the probability of the pattern *x*_*L *- 1 _(*i *- 1) of length *L *- 1 (*x*_*L *- 1 _(*i *- 1) = {*x*(*i *- 1),..., *x*(*i *- *L *+ 1)}) and *p*(*x*(*i*) / *x*_*L *- 1_) the conditional probability of the sample *x*(*i*) given the pattern *x*_*L *- 1_. In (1) the first summation is extended to all the possible *x*_*L *- 1 _patterns, the second one is extended to all the different *L*th samples of the pattern *x*_*L *_(*i*) (*x*_*L *_(*i*) = {*x*(*i*), *x*_*L *- 1 _(*i *- 1)}).

*CE *is maximum if *x *is complex and unpredictable and it reaches zero as soon as a new sample can be exactly predicted from the previous *L *-1 ones.

Using *CE *over short data series can cause an unreliable estimate of *CE *(*CÊ*) : when the conditioning pattern *x*_*L *- 1 _(*i *- 1) is found only once in the series *x *(i.e. *p*(*x*(*i*) / *x*_*L *- 1_) = 1), *CÊ *decreases to zero with *L*. As a consequence both periodic and completely unpredictable signals exhibit *CÊ *equal to zero when *L *increases. Therefore the corrected conditional entropy (*CCE*) must be introduced to perform a reliable measure over short data series:

*CCE*(*L*) = *CÊ*(*L*) + *perc*(*L*)·*Ê*(1)     (2)

where *perc*(*L*) is the percentage of length *L *patterns found only one time in the data set and *Ê*(1) is the estimate of Shannon entropy of the process *x. perc*(*L*)·*Ê*(1) represents the corrective term that compensates the null information associated to the pattern found only once and it increases with *L*, while *CÊ*(*L*) decreases with *L*.

The minimum value of the *CCE *is the best estimate of *CE *and it's taken as an index of complexity: the larger the index, the less predictable the processes. The *CCE *is normalized by the Shannon entropy of the process in order to derive an index independent of the different probability distribution of the processes, thus obtaining:



An index of regularity (the opposite of complexity) may be defined as:

*R*_*x *_= 1 - min(*NCCE*(*L*))     (4)

*R*_*x *_tends to zero if *x *is a fully unpredictable process, it tends to one if *x *is a periodic signal and it assumes intermediate values for those processes that can be partially predicted by the knowledge of the past samples [20].

### Synchronization

The cross-conditional entropy is introduced to define an index of synchronization, related to the repetition of a complex pattern involving two signals.

Given two normalized signals, the cross-conditional entropy of *x *given a pattern *y *is defined as [20]:



where *p*(*y*_*L *- 1_) represents the probability of the pattern *y*_*L *- 1 _(*i*) and *p*(*x*(*i*) / *y*_*L *- 1_) the conditional probability of the sample *x*(*i*) given the pattern *y*_*L *- 1_.

*CE*_*x/y *_represents the amount of information carried by the most recent sample of the signal *x *when *L *- 1 past samples of *y *are known. Over short data series, this definition suffers from the same limitations as conditional entropy, so analogously corrective terms and normalization are introduced. The uncoupling function (*UF*) is defined as:

*UF*_*x,y*_(*L*) = min(*NCCE*_*y/x*_(*L*), *NCCE*_*x/y*_(*L*))     (6)

in order to measure the amount of information carried by one signal that can't be derived from the knowledge of past samples of the other signal. In this way both causal directions are tested and it is taken the one that leads to the best prediction. For every length *L *pattern, *UF *chooses as input the signal that can be the best predictor of the other one. Besides, the joint pattern does not take into account past samples of the output signal to prevent to have a high coupling strength only because one signal has a large index of regularity.

The minimum of *UF *is taken as an index of uncoupling between *x *and *y*, therefore an index of synchronization (the opposite of uncoupling) can be defined as:

*S*_*x,y *_= 1 - min(*UF*_*x,y *_(*L*))     (7)

and it quantifies the maximum amount of information exchanged between the two signals. *S*_*x,y *_tends to zero if the two processes are uncoupled, it tends to one if they are perfectly synchronized and it assumes intermediate values when the two signals are able to exchange a certain amount of information [20].

### Level of predictability

A discrete time series *x*(*n*) can be modeled as the output of an autoregressive model of *p *order



where *n *is the discrete-time index, the *a*_*k *_are the model coefficients and *w*(*n*) is a Gaussian white noise process of variance  feeding the model. The actual sample differs from its model prediction, thus generating the prediction error



An index of the level of predictability (*LP*) may be defined as follows

*LP *= (1 - *σ*_*e *_/ *σ*_*x*_)     (10)

where *σ*_*e *_is the standard deviation of *e*(*n*) and *σ*_*x *_is the standard deviation of the process *x*. *LP *measures the percentage of power which may be predicted by the autoregressive model. In the case of a purely random signal (*σ*_*e *_is quite close to *σ*_*x*_) *LP *tends to zero, while in the case of a linearly predictable signal (*σ*_*e *_tends to zero) the index tends to one and it assumes intermediate values for those processes that may be partially predicted from the model.

### Signal pre-processing

All signals were appropriately recorded and digitized to a 1000 Hz sampling rate at 16-bit resolution. All bipolar electrograms were band-pass filtered (40 – 250 Hz) to remove baseline shift and high frequency noise. In order to cancel the possible effects of ventricular interference (affecting especially recordings during sinus rhythm), the averaged ventricular interference complex was computed and subtracted from each atrial signal [16]. In details, from the surface ECG, the occurrences of QRS were determined, and a template of the ventricular interference in each atrial electrogram was constructed by signal-averaging windows of 140 ms around each QRS (windows were positioned 40 ms before and 100 ms after the R wave). The template was then subtracted from the atrial signals at each occurrence of QRS.

**Figure 2:**
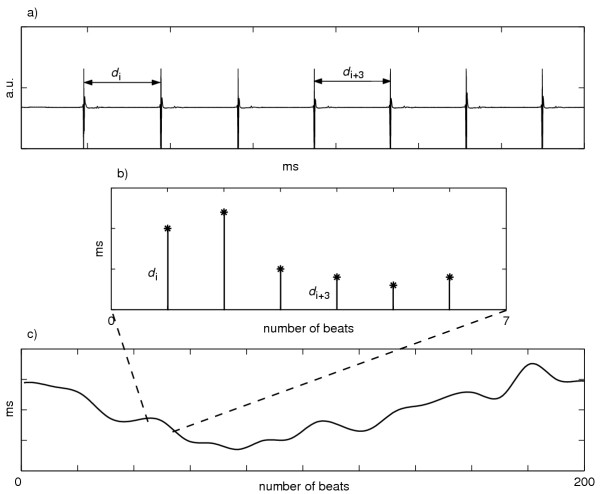
**Extraction of local atrial period (LAP) series from intra-atrial signal. **(a) Example of a recorded electrogram during sinus rhythm before pre-processing; (b)-(c) the related LAP series, obtained as the sequence of temporal distances between two consecutive local atrial activations, after detecting time-instants of local atrial depolarization using a derivative / threshold algorithm. a.u. = arbitrary unit

After detecting time-instants of local atrial depolarization using a derivative / threshold algorithm, the detected depolarizations were visually scored and missed / erroneous detections were corrected by an expert operator using an interactive software. Then, the local atrial period (LAP) series were derived as the sequence of temporal distances between two consecutive local atrial activations [19] (the procedure is shown in Figure 2).

In analogy with previous studies [14,23], after canceling the ventricular interference, the absolute value of the output of the band-pass was low-pass filtered (50 Hz) and then sub-sampled (100 Hz) principally to reduce signal length and computation time.

Considering the number of recorded signals and patients, we analyzed 79 recordings in SR and in SRISO and 77 recordings in AF and in AFISO. Twelve recordings were disregarded for the low quality of electrograms. For each recording, atrial signals were divided into six-second segments and then analyzed. Regularity index was estimated for each six-second segment in each recording site and for each patient, while synchronization index was estimated for each pair of close recording sites (interelectrode distance equal to one).

### Statistical analysis

The statistical analysis was carried out using Student's *t*-test for paired data, comparing each rhythm before and after isoproterenol administration, and between organized (SR) and not organized rhythm (AF).

## Results

### Atrial signals

**Figure 3:**
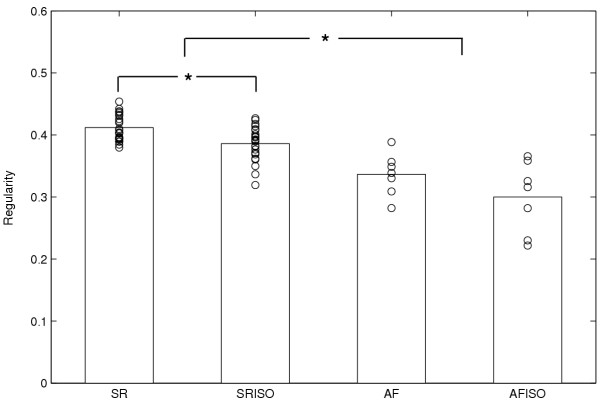
**Values of the R index in a single subject. **Example of regularity (R) index computed for a patient in the electrogram 8 during the four phases of the analysis (SR, SRISO, AF, AFISO). Performance of the R index in the various six-second segments (circle) is superimposed to its mean value. A significant reduction of the R index can be observed passing both from sinus rhythm to atrial fibrillation and from sinus rhythm to sinus rhythm after isoproterenol administration. *p < 0.001

Figure 3 shows an example of the distribution of regularity index (R) computed in one patient and in a single recording site (electrogram 8) during the four experimental conditions. The R values, computed in the six-second segments, are superimposed to their mean value. A significant reduction (p < 0.001) of the index passing from sinus rhythm to AF was detectable. Comparing the results obtained from the same rhythm with and without isoproterenol, a reduction of R was observed after drug administration in both sinus rhythm and atrial fibrillation. In this particular case, the decrease during sinus rhythm was statistically significant (p < 0.001). In particular, considering all patients recording sites, we observed 59 reductions (42 with p < 0.05) over 79 recordings passing from SR to SRISO and 40 (17 with p < 0.05) over 77 passing from AF to AFISO. This result reflects the global tendency of entire dataset, as illustrated in Table 1, where the mean value obtained from all patients recording sites is showed, underlining a statistically significant reduction passing both from SR to AF and from SR to SRISO.

**Table 1:** Mean ± SD values of the proposed indexes in the four analyzed phases

	SR	SRISO	AF	AFISO
AS				
R	0.49 ± 0.08	0.46 ± 0.09^†^	0.29 ± 0.09*	0.28 ± 0.08
S	0.28 ± 0.02	0.28 ± 0.03	0.20 ± 0.06*	0.20 ± 0.06
LAP				
R	0.75 ± 0.07	0.69 ± 0.10^†^	0.31 ± 0.08*	0.26 ± 0.09^†^
LP	99.99 ± 0.001	99.97 ± 0.03^†^	69.46 ± 21.55*	55 ± 24.75^†^

Mean ± SD values of the proposed indexes in the four analyzed phases: sinus rhythm (SR), sinus rhythm during isoproterenol infusion (SRISO), atrial fibrillation (AF) and atrial fibrillation during isoproterenol infusion (AFISO) for Atrial Signals (AS) and LAP series.

^† ^p < 0.0001 the comparison of a same rhythm before and during isoproterenol infusion.

The *t*-test comparing organized (SR) to not organized (AF) rhythms always results significant (* p < 0.0001).

Concerning the synchronization index (S), a significant decrease was observed only when comparing sinus rhythm to atrial fibrillation (Table 1). Evaluating results separately for every recording pair, we observed 35 decreases (7 with p < 0.05) over 69 values passing from SR to SRISO and 32 (9 with p < 0.05) over 66 passing from AF to AFISO.

### Local atrial period

**Figure 4:**
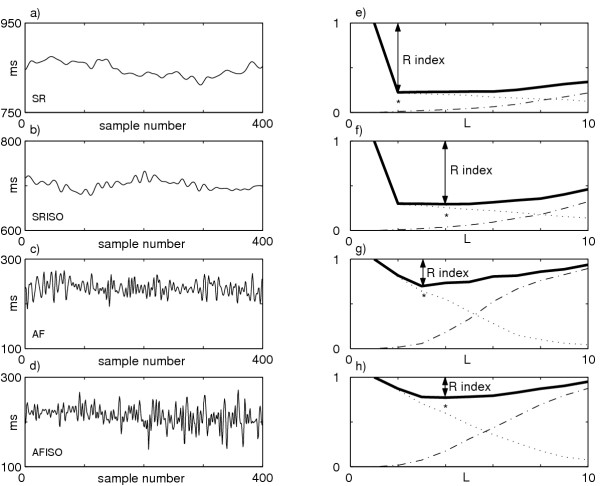
**LAP series in the four analyzed phases and the corresponding *NCCE *functions. **Example of LAP series during (a) SR, (b) SRISO, (c) AF, (d) AFISO; (e)-(h) the corresponding *NCCE *functions (solid lines) depicted as sum of two terms: the decreasing one (CE, dotted line) and the increasing one (the corrective term, dash-dotted line). A clear increase in the minimum value (*) can be observed passing from SR to SRISO to AF to AFISO; therefore the R index = 1 - min(*NCCE*) (see text for more details) decreases passing from SR to SRISO to AF to AFISO.

LAP series were analyzed using the level of predictability and the regularity index. Figure 4 illustrates an example of the LAP series during SR, SRISO, AF, AFISO and the corresponding *NCCE *function. The regularity (*R *= 1 - min(*NCCE*)) decreases visibly passing from sinus rhythm to atrial fibrillation. A decrease in both SR and AF after isoproterenol administration is also observed. The mean values showed in Table 1 are obtained as all patients mean and they underline an analogous tendency. In particular, regularity reductions are found in 7 patients after isoproterenol administration during both sinus rhythm and atrial fibrillation (5 with p < 0.05 passing from SR to SRISO; 2 passing from AF to AFISO).

**Figure 5:**
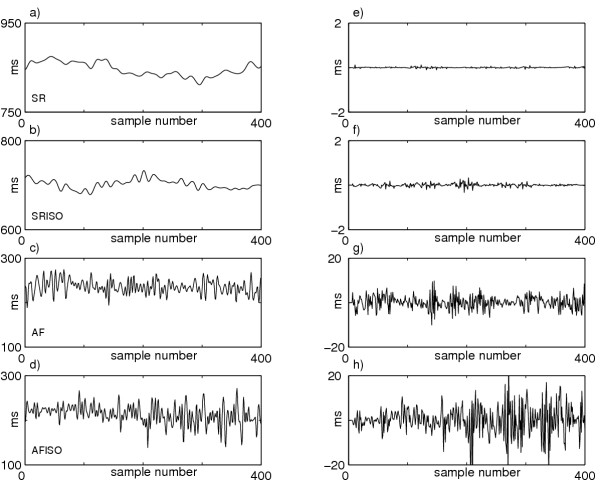
**LAP series in the four analyzed phases and the corresponding prediction errors. **Example of LAP series during (a) SR, (b) SRISO, (c) AF, (d) AFISO; (e)-(h) the corresponding prediction errors. A prediction error increase can be observed passing from SR to SRISO to AF to AFISO, showing the inability of the autoregressive model to predict the LAP series as the rhythm becomes less organized. This is equivalent to a reduction of the LP index = (1 - *σ*_*e *_/ *σ*_*x*_) passing from SR to SRISO to AF to AFISO.

Figure 5 shows the local atrial period series during SR, SRISO, AF, AFISO and the corresponding prediction errors. The prediction error increases passing from SR to SRISO to AF to AFISO. All patients mean reflects this tendency as shown in Table 1. In particular, considering single patients, reductions of level of predictability is observed in 8 of them after isoproterenol administration during both sinus rhythm and atrial fibrillation; the reductions are statistically significant (p < 0.05) in 6 patients passing from SR to SRISO, and only in 4 passing from AF to AFISO.

## Discussion

The autonomic nervous system plays an important role in the genesis and maintenance of atrial fibrillation, but characterization and quantification of its pro-arrhytmic effects are extremely complex and therefore difficult to define. Aim of this study was to evaluate the capability of linear and non-linear parameters to capture the fine changing in the dynamics of atrial signals and LAP series during adrenergic activation induced by the injection of a sympathomimetic drug. The existence of determinism and of an underlining order during AF has recently been shown. In particular both linear [14-16] and non-linear [17,18] patterns have been recognized. In the present study, where a relative small population is considered, we observed a reduction of spatial organization after isoproterenol administration both in sinus rhythm and in atrial fibrillation. In particular analysis of LAP series showed a significant decrease of both LP and R indexes within the same rhythm after isoproterenol administration (see Table 1). This reduction could be related to an increase of atrial wave fronts disorganization. In fact in previous studies [19], both indexes were demonstrated to decrease passing from SR to AF-I and AF-II Wells' classes. Therefore it can be argued that the reduction observed after isoproterenol infusion may be a sign of a high disorganization induced by the drug: atrial activation patterns become less periodic, less predictable and less regular. In fact, in agreement with our previous findings [19], the higher is the regularity and predictability of sequence of atrial activation, the fewer are the circulating 'mother' wavelets according to Jalife's model [24]. This finding is in agreement with known effects of sympathetic activation at atrial level [25] and may provide additional insights to the understanding of the pro-arrhythmic role of ANS in patients with AF. In addition, as previously reported [19], a marked reduction of R and LP indexes was observed passing from SR to AF.

Concerning results obtained from atrial signals, both synchronization and regularity indexes showed a marked reduction passing from SR to AF well in keeping with previous findings [19] that documented the capability of these indexes to discriminate between organized and not-organized rhythms. However the two indexes were not able to evidence any differences after isoproterenol infusion. Only the R index was found significantly decreased in SR after drug administration. Nevertheless, the parameters revealed a tendency toward organization reduction after isoproterenol administration in both rhythms. In particular, responses to isoproterenol were more homogenous during sinus rhythm than during AF. This is maybe due to the fact that patients with a clinical history of AF, could already present alteration in atrial electrical properties likely to be involved in their predisposition to develop AF. Accordingly isoproterenol effects on dynamics of atrial signals are more evident in an organized rhythm (SR) than in AF, where an already disorganized rhythm can not be further fragmented by drug infusion. During AF in fact it has been suggested [1] that several wavefronts of electrical activity propagate through the atria in an irregular manner; this activity may partly obscure isoproterenol effects. Nevertheless, it has also been reported that atrial electrical activity may vary not only in relation to arrhythmia duration but also in relation to the structural characteristics of the atria [26]. In conclusion, the proposed set of linear and non-linear parameters is able to capture subtle changes in atrial dynamics during AF and drug infusion. These indexes could be employed to provide new insight into the mechanisms leading to initiation and maintenance of AF episodes.

## References

[B1] Allessie M, Lammers WJEP, Bonke FIM, Zipes DP, Jalife J (1985). Experimental evaluation of Moe's multiple wavelets hypotesis of atrial fibrillation. In Cardiac electrophysiology and arrhythmias.

[B2] Coumel P, Attuel P, Lavallée JP, Flammang D, Leclercq JF, Slama R (1978). Syndrome d'arythmie auriculaire d'origine vagale. Arch Mal Coeur.

[B3] Liu L, Nattel S (1997). Different sympathetic and vagal effects on atrial fibrillation in dogs: role of refractoriness heterogeneity. Am J Physiol.

[B4] Schauerte P, Scherlag BJ, Pitha J, Scherlag MA, Reynolds D, Lazzara R, Jackman WM (2000). Catheter ablation of cardiac autonomic nerves for prevention of vagal atrial fibrillation. Circulation.

[B5] Inoue H, Zipes DP (1987). Changes in atrial and ventricular refractoriness and in atrioventricular nodal conduction produced by combinations of vagal and sympathetic stimulation that result in a constant sinus cycle length. Circ Res.

[B6] Ashihara T, Yao T, Namba T, Kawase A, Ikeda T, Nakazawa K, Ito M (2002). Differences in sympathetic and vagal effects on paroxysmal atrial fibrillation: a simulation study. Biomed Pharmacother.

[B7] Lombardi F, Tarricone D, Tundo F, Colombo F, Belletti S, Fiorentini C (2004). Autonomic nervous system and paroxysmal atrial fibrillation: a study based on the analysis of RR interval changes before, during and after paroxysmal atrial fibrillation. Eur Heart J.

[B8] Coumel P (1994). Paroxysmal atrial fibrillation: a disorder of autonomic tone?. Eur Heart J.

[B9] Bettoni M, Zimmermann M (2002). Autonomic tone variations before the onset of paroxymal atrial fibrillation. Circulation.

[B10] Dimmer C, Tavernier R, Gjorgov N, Van Nooten G, Clement DL, Jordaens L (1998). Variations of autonomic tone preceding onset of atrial fibrillation after coronary artery bypass grafting. Am J Cardiol.

[B11] Fioranelli M, Piccoli M, Mileto GM, Sgreccia F, Azzolini P, Risa MP, Francardelli RL, Venturini E, Puglisi A (1999). Analysis of heart rate variability five minutes before the onset of paroxysmal atrial fibrillation. Pacing Clin Electrophysiol.

[B12] Tai CT, Chiou CW, Wen ZC, Hsieh MH, Tsai CF, Lin WS, Chen CC, Lin YK, Yu WC, Ding YA, Chang MS, Chen SA (2000). Effect of phenylephrine on focal atrial fibrillation originating in the pulmonary veins and superior vena cava. J Am Coll Cardiol.

[B13] Tai CT (2001). Role of autonomic influences in the initiation and perpetuation of focal atrial fibrillation. J Cardiovasc Electrophysiol.

[B14] Botteron GW, Smith JM (1995). technique for measurement of the extent of spatial organization of atrial activation during atrial fibrillation in the intact human heart. IEEE Trans Biomed Eng.

[B15] Ropella KM (2001). Frequency domain analysis of endocardial signals. Ann 1st Super Sanita.

[B16] Sih HJ, Zipes DP, Berbari EJ, Olgin JE (1999). A high-temporal resolution algorithm for quantifying organization during atrial fibrillation. IEEE Trans Biomed Eng.

[B17] Hoekstra BPT, Diks CGH, Allessie MA, De Goede J (1995). Nonlinear analysis of epicardial atrial electrograms of electrically induced atrial fibrillation in man. J Cardiovasc Electrophysiol.

[B18] Censi F, Barbaro V, Bartolini P, Calcagnini G, Michelucci A, Gensini GF, Cerutti S (2000). Recurrent patterns of atrial depolarization during atrial fibrillation assessed by recurrence plot quantification. Ann Biomed Eng.

[B19] Mainardi LT, Porta A, Calcagnini G, Bartolini P, Michelacci A, Cerutti S (2001). Linear and non linear analysis of atrial signals and local activation period series during atrial-fibrillation episodes. Med Biol Eng Comput.

[B20] Porta A, Guzzetti S, Montano N, Pagani M, Somers V, Malliani A, Baselli G, Cerutti S (2000). Information domain analysis of cardiovascular variability signals: evaluation of regularity, synchronisation and co-ordination. Med Biol Eng Comput.

[B21] Wells JL, Karp RB, Kouchoukos NT, MacLean WA, James TN, Waldo AL (1978). Characterization of atrial fibrillation in man: studies following open heart surgery. Pacing Clin Electrophysiol.

[B22] Porta A, Baselli G, Liberati D, Montano N, Cogliati C, Gnecchi-Ruscone T, Malliani A, Cerutti S (1998). Measuring regularity by means of a corrected conditional entropy in sympathetic outflow. Biol Cybern.

[B23] Barbaro V, Bartolini P, Calcagnini G, Morelli S, Michelucci A, Gensini GF (2000). Automated classification of human atrial fibrillation from intraatrial electrograms. Pacing Clin Electrophysiol.

[B24] Jalife J, Berenfeld O, Skanes A, Mandapati R (1998). Mechanisms of atrial fibrillation: mother rotors or multiple daughter wavelets, or both?. J Cardiovasc Electrophysiol.

[B25] Shimizu W, Tsuchioka Y, Karakawa S, Nagata K, Mukai J, Yamagata T, Matsuura H, Kajiyama G, Matsuura Y (1994). Differential effect of pharmacological autonomic blockade on some electrophysiological properties of the human ventricle and atrium. Br Heart J.

[B26] Gaita F, Calò L, Riccardi R, Garberoglio L, Scaglione M, Licciardello G, Coda L, DiDonna P, Bocchiardo M, Caponi D, Antolini R, Orzan F, Trevi GP (2001). Different patterns of atrial activation in idiopathic atrial fibrillation: simultaneous multisite atrial mapping in patients with paroxysmal and chronic atrial fibrillation. J Am Coll cardiol.

